# Old and New Biomarkers Associated with Endothelial Dysfunction in Chronic Hyperglycemia

**DOI:** 10.1155/2021/7887426

**Published:** 2021-12-27

**Authors:** Maria Pompea Antonia Baldassarre, Caterina Pipino, Assunta Pandolfi, Agostino Consoli, Natalia Di Pietro, Gloria Formoso

**Affiliations:** ^1^Department of Medicine and Aging Sciences, G. d'Annunzio University, Chieti 66100, Italy; ^2^Center for Advanced Studies and Technology (CAST), G. d'Annunzio University, Chieti 66100, Italy; ^3^Department of Medical, Oral and Biotechnological Sciences, G. d'Annunzio University, Chieti 66100, Italy

## Abstract

Chronic hyperglycemia and vascular damage are strictly related. Biomarkers of vascular damage have been intensively studied in the recent years in the quest of reliable cardiovascular risk assessment tools able to facilitate risk stratification and early detection of vascular impairment. The present study is a narrative review with the aim of revising the available evidence on current and novel markers of hyperglycemia-induced vascular damage. After a discussion of classic tools used to investigate endothelial dysfunction, we provide an in-depth description of novel circulating biomarkers (chemokines, extracellular vesicles, and epigenetic and metabolomic biomarkers). Appropriate use of a single as well as a cluster of the discussed biomarkers might enable in a near future (a) the prompt identification of targeted and customized treatment strategies and (b) the follow-up of cardiovascular treatment efficacy over time in clinical research and/or in clinical practice.

## 1. Introduction

Chronic hyperglycemia which characterizes type 2 diabetes (T2D) represent an independent risk factor for cardiovascular diseases (CVD) which are the most common causes of death among subjects with diabetes [1].

There is strong evidence that endothelial dysfunction precedes and fosters atherosclerotic damage thus predisposing to cardiovascular events [2]. Thus, an early detection of vascular damage may allow implementation of strategies able to control CVD development. The progressive loss of endothelial function ensues when the fine balance between circulating elements, blood flow, and endothelial cells is disrupted. Although the mechanisms determining the development of endothelial dysfunction are multiple and complex, in T2D, hyperglycemia plays the leading role [3].

Nowadays, in T2D subjects, the main goal of treatment is not the mere achievement of a satisfactory blood glucose control but the prevention of atherosclerosis and cardiovascular and renal events: this can only be achieved by treatment strategies with an impact on endothelial dysfunction and vascular damage.

This appears to be feasible since data emerging from several clinical trials on cardiovascular outcomes show that use of innovative diabetes drugs, such as glucagon like peptide-1 receptor agonists (GLP1RA) and sodium-glucose cotrasporter-2 inhibitors (SGLT2i), is associated with a reduction in cardiovascular risk in subjects with CVD or at high/very high cardiovascular risk.

However, in order to use in the most appropriate way innovative diabetes treatments, more careful and reliable tools to assess cardiovascular risk are needed. The available techniques for direct evaluation of endothelial function are costly, cumbersome, and time-consuming and require highly specialized personnel, which makes them nonsuitable for use in clinical practice [4, 5]. Moreover, to date, they did not demonstrate sufficient sensitivity and acceptable positive predictive value for cardiovascular risk stratification in patients with T2D.

In such a scenario, several circulating biomarkers might potentially serve as clinical tools enabling a more early and precise evaluation of endothelial dysfunction [6–8]. This is because the endothelium represents the main source for the release of molecules potentially useful as biomarkers for diagnosis, monitoring, pharmacodynamic response, prediction, and prognostic evaluation of CVD.

The purpose of this narrative review is to examine the available data on techniques and biomarkers valuable for detection of hyperglycemia-associated vascular damage in T2D.

Following an overview of classical tools used to investigate endothelial dysfunction, we provide an in-depth description of more promising novel circulating biomarkers (chemokines, extracellular vesicles, and epigenetic and metabolomic biomarkers).

## 2. Materials and Methods

The present narrative review is based on a PubMed literature search including the string “hyperglycemia AND biomarkers”, “diabetes AND biomarkers”, “biomarkers AND vascular AND hyperglycemia”, “peripheral techniques AND vascular AND hyperglycemia”, “inflammatory cytokines AND biomarkers and endothelial dysfunction”, “chemokines AND biomarkers”, “chemokines AND biomarker AND endothelial dysfunction”, “chemokines AND biomarker AND hyperglycemia”, “EVs AND biomarkers AND endothelium AND hyperglycemia”, “epigenetic AND heredity”, “epigenetic AND hyperglycemia AND vascular AND biomarkers”, “epigenetic AND endothelial dysfunction AND hyperglycemia”, and “metabolomics AND vascular AND hyperglycemia”.

The search returned more than 200 articles, which were screened based on the authors' experience to fit the specific aims of this narrative review.

## 3. Assessment of Endothelial Function: Peripheral Techniques

At present, we lack a golden standard technique to assess endothelial function. However, several techniques allow a semiquantitative evaluation of endothelial damage [5]. Among these, plethysmography of the forearm circulation, Flow-Mediated Dilation (FMD), and, more recently, finger plethysmography are more commonly used. All these techniques provide an estimate of endothelial mediated dilation, a phenomenon related to nitric oxide (NO) synthesis, bioavailability, and action. Once produced through the action of the enzyme endothelial nitric oxide synthase (eNOS), NO diffuses to the vascular smooth muscle cells (vSMCs) where it activates the enzyme guanylate cyclase (GC), inducing the production of cyclic guanosine monophosphate (cGMP), a molecule involved in vascular relaxation [9].

Plethysmography of the forearm circulation is a technique used since the early 1900s [10]. It uses plethysmography to measure changes in forearm blood flow in response to vasoactive substances. The technique, which is rather cumbersome, has been used for research purposes only and applied to establish the existence of endothelial dysfunction in subjects with cardiovascular risk factors such as hypercholesterolemia, diabetes mellitus, and cigarette smoking [11, 12].

Being noninvasive, FMD has become the most widely used technique to evaluate endothelial function.

A number of studies identified alterations in FMD as an independent marker of cardiovascular risk [13–15]. This is because FMD provides an index of endothelium-derived NO function, and as such, it might have an independent prognostic value.

This technique needs to be performed by extensively trained operators. Quality of the acquired images, site of measurement, ultrasound probe positioning, and stability of probe positioning throughout the procedure might greatly affect the results. Measurements obtained by self-adjusting ultrasound probes and automatic edge detection of the arterial wall might help in standardizing the techniques and obtaining more reliable results [16, 17]. Moreover, the recent European Society of Cardiology position paper highlighted further weaknesses of the procedure related to the impossibility of standardizing baseline brachial artery diameter [5]. Since the percentage of increase in artery dilation depends on the vessel initial diameter, a situation of basal vasodilation will inevitably result in low value for FMD. This can occur physiologically in a nonstandardized environment (e.g., elevated room temperature), as well as in pathological situations [5]. These considerations impose a great caution in interpreting the true significance of the correlations between altered FMD and clinical outcomes reported in previous prospective studies [13–15]. Nonetheless, assessment of FMD has been the most widely used technique in studies investigating the effect on the endothelium of new drugs for the treatment of T2D subjects [18].

Peripheral arterial tonometry has only recently been introduced as a tool to investigate endothelial function [19]. The analysis of Pulse Wave Amplitude (PWA) aimed at quantifying pulsatile arterial volume changes is obtained by a finger plethysmograph [19].

As a matter of fact, measurement of vascular damage obtained by finger plethysmography seems to associate with several other cardiovascular risk factors, but they not always correlate with FMD data, suggesting that these two measurements provide information on different aspects of vascular damage [20]. Further disadvantages of finger plethysmography are uncertainty about mechanisms explored by the technique (which probably involve nonendothelial factors), lack of reference values, and high cost.

The large majority of studies investigating drug effects on endothelial dysfunction in subject with T2D have used the abovementioned peripheral techniques, i.e., forearm plethysmography, FMD, and finger plethysmography. However, these techniques may not be optimal tools to detect endothelial dysfunction at its earliest stages, mainly because they do not address the initial mechanisms underlying vascular alterations. In addition, as mentioned above, they require highly specialized personnel to be properly performed given the technical challenges they pose [5]. Moreover, although the role of endothelial dysfunction in the pathogenesis of vascular complications is well established, endothelium-dependent peripheral vascular evaluation by peripheral techniques does not appear to improve risk stratification accuracy in patients with T2D [21].

Thus, since peripheral techniques do not seem suitable to detect early functional abnormalities in the vessel wall of people with diabetes, it is worthy to identify functional biomarkers potentially useful to investigate the very early stages of endothelial dysfunction.

## 4. Biomarkers: From the Oldest to the Newest

### 4.1. Inflammatory Cytokines

Inflammation underlies plaque formation and atherosclerosis [22] by inducing endothelial activation and progressive vascular dysfunction. Inflammation indeed diminishes NO availability and alters the balance between endothelium-derived vasodilating and contracting factors [23].

It is not therefore surprising that inflammatory cytokines have been extensively studied as pathogenetic agents and/or as mere biomarkers in atherosclerosis. Inflammatory cytokines have an impact on NO bioavailability and modulate superoxide levels by affecting expression and the activity of both eNOS and NADPH and overall by promoting oxidative stress [24]. Among inflammatory cytokines, increased interleukin-6 (IL-6) levels appear to be associated with increased risk of future cardiovascular events in healthy people and T2D [25].

High-sensitivity C-reactive protein (hsCRP) seems to be a valid cardiovascular risk biomarker. hsCRP is an acute-phase reactant of the pentraxin family released by hepatocytes in response to stimulation by inflammatory cytokines such as interleukin-1 (IL-1), IL-6, and tumor necrosis factor-alpha (TNF-*α*) [26]. Several meta-analysis confirm that hsCRP is an independent predictor of future cardiovascular events even beyond risk score algorithms [27, 28]. However, the prognostic role of hsCRP levels in subjects with T2D is more controversial. Indeed, although Cardoso and colleagues observed an independent association between hsCRP levels and cardiovascular events and mortality in T2D [29], Lowe and collaborators were unable to confirm that hsCRP levels predict macrovascular events and mortality in individuals with T2D with ascertained CVD or cardiovascular risk factors at baseline [30]. The fact that T2D subjects present in general a certain degree of chronic inflammation might be the reason for the poor performance of hsCRP as cardiovascular biomarker in this population [7]. Using inflammatory cytokine levels in combination with information obtained by other techniques for endothelial function assessment might improve the degree of prediction of these biomarkers [5].

### 4.2. Chemokines

In addition to the abovementioned inflammatory markers, interesting evidences show that chemokines might be considered key molecules regulating the pathophysiological process of T2D and its related vascular complications [31]. In fact, once activated by pathogenic stimuli, endothelial cells are able to initiate an inflammatory response in order to recruit immune cells to the site of injury, and chemokines' secretion is one of the first steps of the process [32]. All the other actors of the inflammation, namely, vascular smooth cells (VSMCs), platelets, macrophages, monocytes, and leukocytes, widely express various chemokines and their specific receptors [32]. Chemokines are the largest family of cytokines composed of four subfamilies (C, CC, CXC, and CX3C). They act as signaling molecules which can activate various proinflammatory pathways inducing in turn various inflammatory factors. Among them, a particular interest is deserved to CXCL-12, also known as stromal cell derived factor 1 (SDF-1). It is a CXC chemokine expressed in a variety of tissues where it acts as a potent chemoattractant for progenitor cells [33] accelerating endothelial healing after injury. As a key mediator of regenerative processes, CXCL12 regulates homeostasis, localization, and trafficking of endothelial and smooth muscle progenitor cells through two receptors, CXCR4 and CXCR7 [34]. These two receptors have mutual relationship and interaction with the ligand CXCL12, resulting in the CXCL12/CXCR4/CXCR7 axis which seems to have several roles in atherosclerosis spanning from proatherogenic and prothrombotic to proinflammatory actions modulated by several factors, as reviewed elsewhere [34]. Some clinical observations confirm that the role of CXCL12 per se is proatherogenic in atherosclerosis development and progression. Wurster and collaborators observed that patients with acute coronary syndrome have enhanced platelet surface SDF-1 expression compared to patients with other causes of chest pain [35]. Moreover, increased platelet expression levels of SDF-1 have been found to worsen the clinical outcome in patients with cardiovascular disease [36]. The measurement of CXCL12 expression by the simple flow cytometry method could represent a potential useful biomarker in the very first phases of atherosclerosis. However, more data are needed to confirm the role of CXCL12 and other chemokines as biomarkers in a specific population at high risk for cardiovascular disease, such as T2D subjects.

### 4.3. Extracellular Vesicles

Alternative noninvasive approaches have been developed to study vascular biology's alterations in the peripheral circulation. Emerging cellular markers, namely, cell-derived extracellular vesicles (EVs), are gaining considerable attention.

EVs are 100 nm to 1-micron diameter vesicles produced by virtually all cell types in response to a variety of insults, directly from plasma membrane budding and shedding in different bodily fluids. Smaller vesicles (<100 nm diameter) are usually identified as exosomes. EVs characteristically carry a cargo of bioactive molecules such as microRNA, transcription factors, growth factors, and cytokines, from the related parental cells [37].

EVs are mainly produced and released in response to cell stress and activation following inflammation, oxidative stress, senescence, and many other stimuli [37]. EVs can exert their action as paracrine or remote mediators. EVs originating from endothelium, platelets, and leukocytes seem to be involved in the molecular processes underlying the genesis of atherosclerotic plaque.

Several studies have demonstrated an increase in EVs in subjects with high cardiovascular risk, namely, previous myocardial or cerebrovascular infarction, heart failure, and diabetes [38, 39].

Endothelium-derived EVs (EEVs) seem to reflect endothelial activation and dysfunction in the early stage of the atherosclerotic disease [38]. EEVs are also increased in patients with diabetes and coronary artery disease [40, 41]. Moreover, in patients at high risk of coronary heart disease (CHD), levels of EEVs could be an independent predictor of future development of cardiovascular events and cardiovascular death [42, 43]. Interestingly, Koga and collaborators [41] found that EEVs (EVs CD 144+) were the most significant risk factor for impaired coronary artery dilation in response to acetylcholine in T2D subjects. In addition, some studies have suggested that EEVs carry messages which may influence endothelial cells' reaction to atherogenic insults. Therefore, they could be considered a novel target for the modulation of CVD progression [44, 45]. The paucity of studies and their heterogeneity do not allow to draw clear conclusions on this intriguing hypothesis.

Platelet-derived EVs (PEVs) are altered in several cardiovascular diseases. Patients with carotid plaques or acute atherosclerotic myocardial or cerebral infarction present higher circulating levels of PEVs than control subjects [46–48].

Numerous reports supported the existence of a link between lymphocyte-derived EVs and atherosclerosis. EVs predominantly released by leukocytes (macrophages, lymphocytes, and granulocytes) were detected in atherosclerotic plaques of subjects who underwent carotid endarterectomy [49]. Finally, the observation that circulating leukocyte-derived EVs are predictors of subclinical atherosclerosis burden in asymptomatic individuals further supports their role as a marker of atherosclerosis progression [50].

Altogether, the aforementioned findings suggest that EV shedding relates to atherosclerotic CVD progression.

EVs can also exert paracrine and autocrine actions related to their specific phenotype or their content. As a result of these characteristics, EV levels and phenotype as well as the analysis of their cargo could provide more detailed information on vascular homeostasis and allow to consider EVs as specific biomarkers of endothelial dysfunction.

A subset of EVs exposing phosphatidylserines on the membrane surface is associated with cell death and apoptosis [37]. The possibility to detect the specific EV cargo (microRNA, transcription factors, growth factors, and cytokines) by proteomic analysis could allow the use of EVs not only as biomarkers of endothelial alterations but also as tools to understand the fine mechanisms underlying endothelial dysfunction and to individualize treatment strategies for a wide range of diseases.

Since a number of preanalytical issues may impact EV isolation and could affect the possible translation from basic to clinical research, a plain procedure on fresh peripheral blood samples has been recently developed for identification, enumeration, and separation of EVs from different origins [51].

To date, scarce data are available regarding the effect of drugs for T2D treatment on the modulation of circulating EVs.

### 4.4. Epigenetic Markers

Epigenetics is a branch of genetics that deals with heritable phenotypic changes from a cell or organism, in which no genotype variation is observed [52].

The main epigenetic modifications can be classified as follows: (i) DNA methylation (interferes with the binding of transcription factors to promoters, thus inhibiting gene expression), (ii) modifications of histones (methylation, ubiquitination, phosphorylation, SUMOylation, acetylation, and deacetylation), (iii) remodeling of chromatin, and (iv) noncoding RNA (short interfering RNA, microRNA, piwi-interacting RNAs, and long noncoding RNA) [53, 54].

Over the past decade, an increasing number of studies have revealed an important contribution of epigenetics in vascular dysfunction linked to hyperglycemia [55–58] (Table 1).

It has been shown that early, long-lasting, and persistent exposure to hyperglycemia can leave a footprint in vascular cells that becomes irreversible. This phenomenon is known as “hyperglycemic memory or metabolic memory” [59, 60], following which, despite normalization of blood sugar levels, an altered gene expression due to epigenetic factors persists and is responsible for the progression of micro- and macrovascular diabetes complications [61, 62].

The first evidence of this came from a 1980 study aimed at comparing incidence of diabetic retinopathy in dogs kept at hyperglycemia for 2.5 years and then returned to normal blood glucose levels versus nondiabetic and diabetic dogs [63]. Unexpectedly, this study revealed that the incidence of retinopathy in the first group was comparable to that observed in diabetic dogs. During the last decades, a host of studies, using both *in vivo*, ex vivo, and *in vitro* models, have focused on unravelling epigenetic mechanisms, in the attempt to elucidate the molecular bases of this phenomenon [64–70]. Several studies have been conducted on endothelial cells, since these are the first to be exposed to blood glucose level changes leading to endothelial dysfunction [54, 57, 65, 68, 69, 71–77]. In this regard, we have previously shown that endothelial cells derived from the umbilical cord of Caucasian mothers with gestational diabetes (GD-HUVECs) and therefore exposed *in vivo* to elevated glucose levels maintained a “diabetic cardiovascular phenotype” characterized by inflammatory imbalance associated with insulin resistance, increased levels of superoxide anion, and reduced NO availability [78, 79] even after long exposure to normal glucose levels *in vitro*. This might be interpreted as the result of a sort of epigenetic control of gene expression [76, 78]. It has also been shown that glucose-induced *in vitro* upregulation of oxidative stress markers persists in HUVECs exposed to a constant high glucose concentration long after glucose normalization, a phenomenon defined by the authors as “endothelial hyperglycemic memory” [58, 80, 81].

On this basis, it appears evident that the epigenetic studies of endothelial dysfunction associated with hyperglycemia undoubtedly represent a promising strategy for quantitative as well as qualitative vascular damage detection.

Among epigenetic modifications, DNA methylation and posttranslational histone modifications are the most prominently involved in hyperglycemic memory. Histone methyl transferase (HMT) Set7 is overexpressed in hyperglycemic human aortic endothelial cells (HAECs) or in diabetic peripheral blood cells, and it is directly related to increased activation of the NF-*κ*B p65 proinflammatory gene promoter, even after restoration of normoglycemic conditions [82]. Interestingly, silencing of Set7 prevented DNA methylation and NF-*κ*B-mediated proinflammatory signaling cascade activation [82, 83]. This observation suggests that Set7 inhibitors could potentially be designed to erase hyperglycemic memory and mitigate diabetic vascular complications [84]. Furthermore, an interesting recent study has provided new evidence that hyperglycemia triggers dose-responsive changes in DNA methylation dynamics that influence the key physiological processes involved in the maintenance of endothelial function [77].

Regarding histone acetylation and deacetylation through histone acetyltransferase (HAT) and histone deacetylase (HDAC), respectively, hyperacetylation of histone H3K9/K14 and subsequent upregulation of genes involved in metabolic and cardiovascular diseases have been observed in primary vascular endothelial cells exposed to hyperglycemia [57]. Sirtuins are HDACs acting on specific endothelial targets, able to regulate various processes, including inflammation, expression of cytokines (IL-6, TNF-*α*, NF-KB, and MMP-14), oxidative stress (manganese superoxide dismutase (MnSOD), FOXOs), and deacetylation of histone H3K14 and H4K16 [85]. High glucose concentration was found to induce endothelial cell senescence and functional abnormalities through repression of sirtuin 1 (SIRT1). Interestingly, in the same cellular model, SIRT1 overexpression appears to be protective against glucose-induced endothelial dysfunction, thus potentially preventing diabetic vascular complications [86]. Furthermore, Mortuza and colleagues showed that glucose-induced downregulation of SIRT1 was accompanied by reduced levels of FOXO1-mediated antioxidant enzymes, suggesting that the SIRT1/FOXO1 axis is able to regulate the oxidative state in endothelial cells [85].

The described hyperglycemic-associated histone modifications could potentially contribute to the upregulation and downregulation of a variety of miRNAs that, on the basis of the regulatory pathways they are involved in, could be generally grouped into angiogenesis-associated miRNAs (angioMiRs), inflammation-associated miRNAs (inflammaMiRs), and senescence-associated miRNAs (seneMiRs) [87].

The role of these specific groups of miRNAs in endothelial function/dysfunction associated with hyperglycemia has recently been thoroughly reviewed by Coco and collaborators, who identified and classified the main miRNAs involved in hyperglycemia-induced endothelial dysfunction, including angioMiRs miR-126, miR-483-3p, miR-106, and miR-342-3p; inflammaMiRs miR-155, mir-126, miR-23b-3p, and miR-200c; and seneMiRs miR-146a [58].

Elevated levels of miR-155 lead to increased endothelial permeability with consequent macrophage infiltration, thus promoting plaque formation and atherosclerosis progression [88, 89]. Like other inflammaMiR, miR-155 levels correlate with glucose concentrations endothelial cells are exposed to. On the contrary, it has been shown that miR-126 reduction leads to enhanced NF-*κ*B activation, TNF*α* and VCAM1 expression, and consequent increase in leukocyte adhesion [90, 91]. In addition, miR-126, also belonging to angioMiRs, has been shown to regulate angiogenesis and vascular integrity by inhibiting the vascular endothelial growth factor- (VEGF-) mediated signaling pathway [92].

Some miRNAs play a key role in the mechanisms underlying oxidative stress-induced senescence. Among these, miR-146a regulates NADPH oxidase 4 (NOX-4) expression and Reactive Oxygen Species (ROS) production in endothelial cells [93].

Interestingly, ROS can in turn activate miRNAs, which in a complex network can modulate both histone methylation and acetylation/deacetylation [94]. Among ROS-activated miRNAs, miR-200c, belonging to the miR-200 family, promotes endothelial cell apoptosis and senescence. At the same time, high levels of miR-200c disrupt the SIRT1/FOXO1/eNOS loop which plays a key role in vascular homeostasis [94, 95]. As a matter of fact, an abnormal miR-200c overexpression increases p66Shc phosphorylation, which consequently contributes to increased oxidative stress as well as vascular alterations [94, 96]. Finally, in endothelial cells, it has been observed that hyperglycemia is capable of upregulating or downregulating the long coding RNAs (IncRNAs) [97]. Although most lncRNAs contribute to endothelial dysfunction, some of them, namely, lncRNA-MEG3, might have a positive role as a stimulator of the antiatherogenic PI3K/Akt signaling pathway [98].

Overall, the epigenetic changes described, together with a host of other epigenetic factors [99], therefore suggest their potentially relevant role in the onset and progression of vascular complications.

Furthermore, it is important to underline that DNA methylation and miRNAs are the main determinants involved in the diabetic “interactome” [100]. Hence, identifying epigenetic interactions between miRNA and DNA methylation associated with gene expression can help advance our knowledge about the molecular mechanisms underlying hyperglycemia-induced vascular damage. This could also foster their clinical use as predictive biomarkers of cardiovascular complications or even make them potentially useful to furtherly develop “precision medicine” in the context of diabetes.

### 4.5. The Metabolomic Approach

Given the pressing need of identifying endothelial dysfunction at a very early stage in order to prevent disease progression, novel original biomarkers need to be sought by means of innovative technologies [101]. Therefore, in the latest years, research on vessel damage biomarkers has focused on the application of unbiased methods to phenotype disease. The advent of new technologies such as microarrays, RNA sequencing, and mass spectrometry (the so-called “omics”) has generated thousands of data, leading to the identification of a number of genes, pathways, RNAs, and metabolites potentially involved in diseases [102].

Regarding T2D and its vascular complications, the complexity of metabolic changes characterizing these diseases poses a formidable challenge to an exhaustive understanding of the involved molecular pathways. In this context, combining different omics strategies used for the global profiling of genome, transcriptome, miRNA-ome, DNA methylome, and metabolome could help in defining endothelial dysfunction and atherogenesis biological and pathophysiological mechanisms in chronic hyperglycemia and in identifying new potential predictive biomarkers (Table 1) [103].

In particular, the metabolome, the terminal product downstream from genome, transcriptome, and proteome including the whole panel of small-molecule intermediates and products of cellular metabolism, represents a new platform able to offer a comprehensive estimate of metabolite changes occurring within a cell, tissue, or body fluid in response to genetic alterations, pathophysiological stimuli, and environmental factors. Targeted metabolomics is based on the addition to the sample of stable isotope-labeled standards prior to extraction, in order to quantify absolute concentrations of known metabolites. Conversely, untargeted metabolomic approaches can detect and quantify concentrations of a broad spectrum of metabolites by measuring the differences between two experimental conditions or groups of patients [104]. Metabolomic technology, commonly based on mass spectrometry (MS) and/or nuclear magnetic resonance (NMR) techniques, is becoming more and more used in biomarker research because it can be integrated with genomics, transcriptomics, and proteomics. Thus, coupled with the identification of biological phenotypes, it can improve our understanding of disease metabolic bases, and it can help in shedding light on pathogenesis as well as on metabolic impact of diseases and their progression [105, 106].

Indeed, perturbations in plasma amino acid and/or lipid metabolism identified by metabolomic have been associated with the risk of developing T2D, suggesting a potential role of these changes in the pathogenesis of diabetes and the possibility of using them as targets for diabetes prevention strategies [107, 108].

Furthermore, among subjects with T2D, individuals presenting microalbuminuria showed a different metabolomic profile as compared to subjects without microalbuminuria. In detail, lower levels of plasma histidine and higher levels of butenoylcarnitine were detected in patients with microalbuminuria as compared to controls. Moreover, hexose, glutamine, and tyrosine excretion was lower in urine samples of subjects with microalbuminuria as compared to controls. These metabolites, together with established kidney disease risk markers may help in predicting macroalbuminuria development in T2D patients [109].

Alterations in the metabolomic profiles were observed in a study performed to evaluate aortic metabolic content in diabetic mice with hyperglycemia, hyperlipidemia, hypertension, and stenotic vascular damage. Several metabolite changes were found in pathways related to carbonyl stress, carbohydrate metabolism, and amino acid metabolism. Interestingly, three original pathways involving vitamin B6, propanoate, and butanoate metabolism were discovered associated with diabetic vascular complications [110].

Metabolomic analyses also demonstrated significant alterations in alanine, proline, glycine, serine, and glutamine in human aortic endothelial cells exposed to acute and chronic hyperglycemia, thus indicating that amine levels change in high glucose conditions in a time-dependent manner [111].

In addition, a study in hyperglycemic mice identified the glycosphingolipid pathway as a potential therapeutic target to prevent or slow down atherogenesis in diabetes [112].

In the latest years, genome-wide association studies (GWAS) have also been used to examine the impact of genetic variation on plasma metabolites. A study in endothelial cells carrying a Single Nucleotide Polymorphism (SNP) associated with CHD in T2D [113] and showing lower glutamine synthase activity identified altered intracellular metabolic traits, including impairments of the *γ*-glutamyl cycle and methylglyoxal detoxification [114]. The results of this metabolomics-based study suggest that glutamine supplementation could be explored as a potential novel approach for CHD prevention in T2D patients carrying the SNP. Not only pathologies and genetic factors but also environmental factors such as diet, physical activity, medication, and microbiome may influence the metabolome [115]. A recent metabolomic study investigated the effect of aspirin eugenol ester on vascular endothelial dysfunction in atherosclerotic rats and HUVECs exposed to hydrogen peroxide. In these models, aspirin eugenol ester altered amino acid, carbohydrate, energy, and vitamin metabolism. In particular, levels of an l-methionine intermediate metabolite inhibiting the apoptosis of vascular endothelial cells were markedly increased by exposure to the drug [116]. Thus, by investigating the levels of endogenous metabolites through a sensitive metabolomic approach, it is possible to detect alterations in metabolic pathways in response to various stimuli [117]. Hence, although application of the omics approach to the study of the relationships between hyperglycemia and early endothelial dysfunction is still premature, data gathered by metabolomics in this field are already providing novel insights into the development of atherosclerosis [104]. Indeed, these technologies represent innovative risk assessment tools and support the notion that diabetes vascular complications arise from the interplay of thousands of metabolic pathways together with oxidative stress. Dissecting these by metabolomics might provide not only new and much needed biomarkers but also insights into novel therapeutic targets. For this to be accomplished, however, and to achieve a better prediction of future risk and disease progression, one needs to correlate biomarkers found in conditions of hyperglycemia with early endothelial dysfunction. Thus, future in-depth, large-scale, high-throughput, “omics” studies are needed to define new biomarkers and therapeutic targets at an early stage of disease to be able to prevent/slow down the atherosclerosis process.

## 5. Limitations of Current and Novel Biomarkers

As reported in a recent Position Paper on behalf of the European Society of Cardiology (ESC), “the ideal method to assess endothelial function should be noninvasive, easy to use, prospectively validated in different cohorts and ethnic groups, with an incremental value over standard, clinically established risk markers, cost-effective, measured according to methodological consensus and providing reference values as a basis for treatment” [5]. Although each of the biomarkers covered in this narrative review has important potential, none of them possess all these characteristics.

Approaches based on the use of physical devices (such as plethysmography or FMD), so far widely used to detect vascular alteration, present several limitations hampering their use and making it difficult to interpret their results: (a) they are cumbersome techniques depending on operator skills, (b) differences in the techniques and in the methods used by different investigators make it difficult to directly compare results obtained in different studies, and (c) they do not provide information on vascular pathogenic mechanisms.

Circulating biomarkers may fill the gaps in available techniques because they are directly enlisted or secreted by endothelial cells.

Nevertheless, inflammatory cytokines demonstrated a poor performance as cardiovascular biomarkers in T2D subjects [7], and chemokines need yet to be tested as biomarkers for the prediction of CV events in this specific population. EVs are very promising since they could serve as biomarkers as well as mediators of vascular alteration. Unfortunately, a consensus about the methods to be used for their detection and enumeration is not yet internationally been reached.

At present, the mechanisms that can link epigenetic changes to endothelial dysfunction in hyperglycemia in a cause-effect relationship are far from being unambiguously elucidated. It is important to emphasize that epigenetic changes can be species-specific, and this should be carefully considered when translating epigenetic results from animals to humans. Furthermore, currently epigenetic strategies in the clinical setting are complicated by several reasons such as the ability of a single noncoding RNA to regulate different biological processes, mRNA-lncRNA interactions in the vascular system, and the high toxicity of experimental molecules.

Ongoing research in this area will provide useful information to identify and monitor specific epigenetic mechanisms related to endothelial dysfunction in diabetes.

Regarding the metabolomic approach, although there are significant advancements in generating many tools for analysing and interpreting the massive amount of metabolomic data, there are still some limitations in the translation of research findings. Studies so far available on the metabolomic approach are mainly focused on developing accurate methods for metabolite measurements and data interpretation. Moreover, most of the studies are focused on biomarkers related to hyperglycemia but lack the correlation with endothelial dysfunction which would allow to achieve a better prognostic meaning.

Another aspect that makes translational metabolomics difficult is experimental costs, particularly the cost of analytical instrumentation. Thus, efforts in making this technology less expensive and accessible should be encouraged.

Finally, more research should be carried out with the aim of integrating metabolomic data with other omics data to establish a platform for multiomics integration.

## 6. Conclusions

Biomarkers of vascular damage have been intensively studied in the recent years in the quest of reliable cardiovascular risk assessment tools able to facilitate endothelial dysfunction assessment.

**Figure 1:**
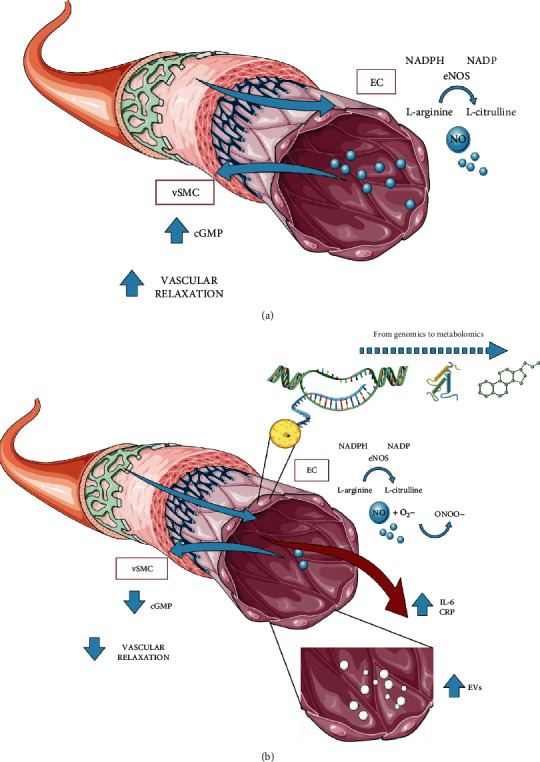
Graphic representation of the mechanisms underlying endothelial function (a) and dysfunction (b). (b) Indicates the old (IL-6, CRP) and emerging (EVs, genomics, and metabolomics) biomarkers of vascular damage assessment. Nitric oxide (NO) production plays a central role in modulating endothelial function. NO is generated from the metabolism of l-arginine by the endothelial nitric oxide synthase (eNOS). In an inflammatory and prooxidant environment, as occurs in diabetes, the superoxide anion may quench NO, thereby reducing the efficacy of a potent endothelium-derived vasodilator system that participates in the general homeostasis of the vasculature (b). The consequent altered endothelial homeostasis is associated with the production of molecules involved in vascular damage and atherosclerotic plaque progression. vSMC: vascular smooth muscle cells; cGMP: cyclic guanosine monophosphate; EC: endothelial cell; NADPH: nicotinamide adenine dinucleotide phosphate (reduced form); NADP: nicotinamide adenine dinucleotide phosphate; eNOS: endothelial nitric oxide synthase; NO: nitric oxide; O_2_-: superoxide anion; ONOO-: peroxynitrite; IL-6: interleukin-6; CRP: C-reactive protein; EVs: extracellular vesicles.

Innovative emerging biomarkers might prove themselves instrumental in defining individual phenotypes, in identifying vascular disease stages and progression, in detecting changes induced by drug intervention, and, eventually, in devising new treatment strategies. This is because these molecules/vesicles (as shown in Figure 1) seem to be directly involved in the mechanisms leading to vascular damage and represent sensible indicators of early endothelial metabolic disturbance signs [5].

Although potential biomarkers useful for a qualitative and quantitative vascular damage characterization have been identified, it is necessary to attribute to these markers the right value and target of use in research and clinical practice. Specific studies are needed in order to characterize each marker and to elucidate the information that it can provide in terms of predictive, diagnostic, prognostic, and monitoring value [118].

Based on the wealth of information that omics sciences can provide, performing CV markers could be identified through this technology. A validated interpretation of the big amount of data and their appropriate application may hopefully lead in the near future to the so-called integrated Personalized Omics Profiling (iPOP), opening the way to an efficient precision medicine able to provide personalized disease prevention and treatment plans [119–121].

Proper use of a single as well as a cluster of the biomarkers discussed above might enable (a) the prompt identification of targeted and customized treatment strategies and (b) the follow-up of cardiovascular treatment efficacy over time in clinical research and/or in clinical practice.

## Figures and Tables

**Table 1:** The table summarizes potential epigenetic and metabolomic biomarkers associated with endothelial dysfunction in chronic hyperglycemia.

Technology	Biomarkers and/or pathway	Source type	References
*Epigenetics*	HMT Set7 overexpression and NF-*κ*B p65 activation	Human aortic endothelial cells (HAECs) exposed to high glucose and aortas of diabetic mice	[82]
	Hyperacetylation of histone H3K9/K14	Human aortic endothelial cells (HAECs) in high glucose or low glucose	[57]
	SIRT1 overexpression	Endothelial cells (ECs) and tissues from diabetic male C57BL/6 mice	[85, 86]
	Angiogenesis-associated miRNAs (angioMiRs), inflammation-associated miRNAs (inflammaMiRs), and senescence-associated miRNAs (seneMiRs)	Endothelial cells (ECs); human dermal microvascular endothelial cells (HMVECs); human umbilical vein endothelial cells (HUVECs); vascular smooth muscle cells (VSMCs)	[58, 87]
	miR-155	Endothelial cells (ECs); vascular smooth muscle cells (VSMCs); female Apoe–/– mice	[88, 89]
	miR-126	Plasma of sepsis patients and healthy controls; RAW264.7 macrophages; human umbilical vein endothelial cells (HUVECs); human promyelocytic cell line HL-60; endothelial cells derived from mouse embryonic stem (ES) cells; knockdown of miR-126 in zebrafish	[90–92]
	miR-146a	Human umbilical vein endothelial cells (HUVECs)	[93]
	miR-200c	Endothelial cells (ECs); human umbilical vein endothelial cells (HUVECs); C2C12 myoblasts; primary normal human fibroblasts	[94, 95]
	IncRNAs	Human umbilical vein endothelial cells (HUVECs) cultured under high or normal glucose conditions	[97]
	IncRNA-MEG3	Retinas of STZ-induced diabetic mice; endothelial cells (ECs)	[98]

*Metabolomics*	Low plasma histidine, high butenoylcarnitine Low hexose, glutamine, and tyrosine	Urine and plasma of T2D patients	[109]
	Alterations of carbonyl stress, carbohydrate metabolism, amino acid metabolism pathways, in particular vitamin B6, propanoate, and butanoate metabolism	Mouse aortic cells	[110]
	Alanine, proline, glycine, serine, and glutamine alterations	Human aortic endothelial cells (HAECs) exposed to acute and chronic hyperglycemia	[111]
	Glycosphingolipid pathway	Plasma of hyperglycemic mice and glucosamine-supplemented mice	[112]
	Gamma-glutamyl cycle impairment and increased methylglyoxal	Human umbilical vein endothelial cells (HUVECs)	[114]
